# Induction of Broad Immunity against Invasive Salmonella Disease by a Quadrivalent Combination Salmonella MAPS Vaccine Targeting Salmonella Enterica Serovars Typhimurium, Enteritidis, Typhi, and Paratyphi A

**DOI:** 10.3390/vaccines11111671

**Published:** 2023-10-31

**Authors:** Emily M. Boerth, Joyce Gong, Becky Roffler, Claudette M. Thompson, Boni Song, Sasha F. Malley, Angelika Hirsch, Calman A. MacLennan, Fan Zhang, Richard Malley, Ying-Jie Lu

**Affiliations:** 1 Division of Infectious Diseases, Boston Children’s Hospital, Harvard Medical School, Boston, MA 02115, USA; 2 Enteric & Diarrheal Diseases, Global Health, Bill & Melinda Gates Foundation, 500 5th Ave. N, Seattle, WA 98109, USA

**Keywords:** Salmonella, MAPS vaccine, OSP, SseB, quadrivalent, Typhimurium, Enteritidis

## Abstract

Bloodstream infections in low- and middle-income countries (LMICs) are most frequently attributed to invasive Salmonella disease caused by four primary serovars of *Salmonella* enterica: Typhi, Paratyphi A, Typhimurium, and Enteritidis. We showed previously that a bivalent vaccine targeting *S*. Typhi and *S*. Paratyphi A using a Multiple Antigen-Presenting System (MAPS) induced functional antibodies against *S*. Typhi and *S*. Paratyphi. In the current study, we describe the preclinical development of a first candidate quadrivalent combination *Salmonella* vaccine with the potential to cover all four leading invasive *Salmonella* serotypes. We showed that the quadrivalent *Salmonella* MAPS vaccine, containing Vi from *S*. Typhi, O-specific Polysaccharide (OSP) from *S.* Paratyphi A, *S.* Enteritidis and *S.* Typhimurium, combined with the *Salmonella*-specific protein SseB, elicits robust and functional antibody responses to each of the components of the vaccine. Our data indicates that the application of MAPS technology to the development of vaccines targeting invasive forms of Salmonella is practical and merits additional consideration.

## 1. Introduction

Invasive *Salmonella* disease is the most common cause of blood stream infections in low- and middle-income countries (LMICs), but it is much more than just typhoid; it encompasses enteric fever (typhoid and paratyphoid A) and invasive nontyphoidal *Salmonella* disease. Four key serovars of *Salmonella enterica* are responsible for a large majority of invasive *Salmonella* disease: *Salmonella enterica* serovars Typhi, Paratyphi A, Typhimurium, and Enteritidis. Invasive nontyphoidal *Salmonella* (iNTS) disease has a high associated case fatality rate and particularly affects infants and immunocompromised individuals in sub-Saharan Africa. Although conjugate vaccines against Salmonella Typhi are licensed and World Health Organization (WHO)-prequalified, there are currently no such vaccines for invasive non-typhoidal Salmonella (iNTS) disease and paratyphoid A. This study outlines the preclinical development of the first candidate quadrivalent combination Salmonella vaccine, aiming to cover all four major invasive Salmonella serotypes. The focus is on generating components against *S*. Typhimurium and *S*. Enteritidis to complement the components against *S*. Typhi and *S*. Paratyphi A described in our previous report [1].

iNTS disease is caused predominantly by serovars *Salmonella enterica*, serovar Typhimurium, and *Salmonella enterica* serovar Enteritidis [2]. Globally, over 70% of all human isolates of iNTS are *S*. Typhimurium and *S*. Enteritidis [3,4]. Poor sanitation and water treatment are frequently the cause of fecal–oral transmission of iNTS. In children under 5 years old, 12–28% of the incidence of iNTS disease is fatal [5]. There is therefore an urgent need for a vaccine targeting iNTS, particularly for pediatric populations. Thus, the development of a vaccine against both *S.* Typhimurium and *S.* Enteritidis holds the potential to substantially decrease the morbidity and mortality associated with iNTS disease.

One proposed vaccine strategy is to generate antibodies to polysaccharide components of the organisms. *Salmonella* Typhi has a capsular polysaccharide, Vi, which is the main component of seven licensed and WHO-prequalified conjugate vaccines against the organism [6]. While equivalent capsular polysaccharides are not present on other serovars of *Salmonella enterica*, there are O-specific polysaccharides (OSPs) on the surface of the latter which are components of lipopolysaccharide. An OSP of *S*. Paratyphi is being evaluated as a vaccine candidate [6]. *S*. Typhimurium and *S*. Enteritidis have unique O-specific polysaccharides that elicit antibody-mediated bacterial clearance [7,8,9,10,11].

In addition, genetically conserved proteins on the bacterial surface have also been proposed as potential vaccine targets, several of which are being studied preclinically. One antigen of particular interest is the serodominant secreted effector protein, SseB [12,13,14]. SseB is genetically conserved across *Salmonella* species and localized to the bacterial surface [13,15,16]. SseB has been used in a candidate subunit vaccine to protect chickens from infection [16,17].

In the present studies, we describe the use of a platform vaccine technology called MAPS (for Multiple Antigen-Presenting System), which, by combining both OSP and SseB, may offer important immunological advantages for protection against *Salmonella*. MAPS combines a rhizavidin-containing fusion protein antigen and a biotinylated polysaccharide to generate an immunogenic complex [18]. A 24-valent MAPS vaccine against *Streptococcus pneumoniae* has successfully completed Phase 1/2 clinical trials, attesting to the tolerability and immunogenicity of the platform [19] (NCT03803202). We have also previously demonstrated that a MAPS vaccine consisting of the Vi capsular polysaccharide for *S.* Typhi and the O-polysaccharide of *S.* Paratyphi A generates functional immune response [1]. This work led us to study MAPS complexes targeting *S.* Typhimurium and *S.* Enteritidis. We present here different MAPS complexes using a *Salmonella* specific protein, SseB, and OSP from *S.* Enteritidis J73 and *S.* Typhimurium S12. We evaluated the protective function of antibodies generated by MAPS and demonstrated that these *Salmonella* MAPS complexes generate functional antibodies in different formulations. We show that a quadrivalent *Salmonella* MAPS vaccine, containing OSP from *S.* Paratyphi A, *S.* Enteritidis, *S.* Typhimurium and *S*. Typhi, Vi, combined with SseB, elicits robust and functional antibody responses to each of the components of the vaccine.

## 2. Materials and Methods

Materials: Aluminum phosphate (alum) was obtained from Brenntag North America (Reading, PA, USA). Vi polysaccharide was provided as a gift by Dr. Szu from the NIH [20]. Adipic acid dihydrazide (ADH), 1-Ethyl-3-[3-dimethylaminopropyl] carbodiimide Hydrochloride (EDC), N-hydroxysulfosuccinimide (NHS), and 1-cyano-4-dimethylaminopyridinium tetrafluoroborate (CDAP) were purchased from Thermo Fisher (Waltham, MA, USA). Restriction endonucleases and T7 shuffle expressing competent cells were purchased from New England Biolabs (Ipswich, MA, USA). Plasmid pETDuet and other reagents were obtained from Sigma (St. Louis, MO, USA). iNTS strains were obtained from the Global Enteric Multicenter Study collection through Dr. Sharon Tennant at the University of Maryland.

Protein Purification. DNA fragments encoding His-SseB and SseA (the chaperone protein for SseB) or His-IpaB and IpgC (the chaperone protein for IpaB) were synthesized and inserted into a pETDuet vector containing Rhavi through restriction enzyme digestion and ligation. Plasmids confirmed by sequencing were introduced into *E. coli* T7 shuffle express cells. Transformants with the respective cloned proteins were cultivated to OD600 = 1 at 25 °C, followed by induction of protein expression with 0.2 mM IPTG at 16 °C overnight. After centrifugation, cell pellets were resuspended in lysis buffer (20 mM Tris-HCl, 500 mM NaCl, pH 8.0) and then sonicated to lyse. The supernatant was passed over a Ni-NTA column, and SseA or IpgC was washed off the column with 0.05% LDAO in a lysis buffer. Rhavi-SseB and Rhavi-IpaB proteins were then eluted in a buffer containing 250 mM Imidazole. Elutions containing the proteins were combined and subjected to purification over a gel-filtration column to isolate dimer fractions.

Purification of OSP from *S*. Paratyphi, *S*. Typhimurium, and *S*. Enteritidis. GMP-grade Vi was purchased from Walvax Inc. (Kunming, China). OSPs were purified from *S*. Paratyphi 9150 (ATCC) and clinical strains of *S*. Typhimurium and *S*. Enteritidis using a protocol established previously, with modifications [21]. Briefly, bacteria were resuspended in 6% acetic acid and boiled for 4 h at 100 °C. Supernatant was extensively dialyzed against water and then lyophilized. Resuspended OSP was treated with Q cassette and ethanol precipitation, dialyzed, and then loaded on a gel-filtration column to separate the OSPs with larger sizes. The final OSP was lyophilized to concentrate and frozen in −20 °C. Concentration was determined by the Anthrone method [22].

Biotinylation of OSP. *S.* Typhimurium, *S.* Enteritidis, and *S.* Paratyphi A OSPs were biotinylated with CDAP using a protocol described previously [18]. Briefly, OSP was diluted to a concentration of 2 mg/mL and incubated while stirring with CDAP (100 mg/mL) for 30 s and then with 50 mM Sodium borate for 2 min. Amine-PEG3-biotin (40 mg/mL) was added at a ratio of 1:1 (*w*:*w*) and stirred for 2 h at room temperature. Reaction was terminated by the addition of glycine to 20 mM final concentration. Biotinylated OSP underwent extensive saline dialysis before its utilization in MAPS assembly. Vi was biotinylated using Amine-PEG3-Biotin, following a previously described method, with slight modifications [18]. In summary, Vi was reconstituted to a concentration of 5 mg/mL in buffer A (0.2 M MES, 150 mM NaCl, pH 5.8). EDC (100 mg/mL in buffer A) and NHS (100 mg/mL in Dimethylformamide) were added to the solution and allowed to react for 15 min at room temperature. The solution’s pH was adjusted to pH 7.0 by adding 1 M NaHCO3 (pH 10). Amine-PEG3-Biotin (40 mg/mL in water) was introduced in a 1:1 (*w*:*w*) ratio. The reaction continued to be stirred for an additional 2 h at room temperature before the addition of glycine to achieve a final concentration of 20 mM. Biotin concentration was determined by a No-Weigh HABA/avidin Premix Biotin Quantification Kit. Vi PS was determined by acridine orange assay. Briefly, samples and standard (0.4 mg/mL) were serially diluted. Acridine orange was diluted to 1:10 dilution, added to each sample, and read at 490 nM.

MAPS formulation. All MAPS were assembled by rotating the relevant components at 4 °C overnight and purifying via size-exclusion chromatography. *S*. Typhimurium and *S*. Enteritidis MAPS were assembled at a 4:1 (*w*:*w*) protein: polysaccharide ratio and purified with size-exclusion columns. Vi MAPS were assembled at a 2:1 ratio and ParaOSP MAPS were assembled at a 6:1 ratio. Protein concentration was determined by the BCA method (Pierce), and OSP concentration was determined using the anthrone method [22]. Vi PS concentration was determined by acridine orange assay. A MAPS complex with one protein type and one polysaccharide type was considered a monovalent MAPS. Multiple monovalent MAPS could be combined into one immunization to generate immune responses against multiple *Salmonella* serovars and were referred to as bivalent and quadrivalent MAPS.

Antigen preparations and immunization procedures. In MAPS immunization experiments, vaccines were combined with aluminum phosphate (alum) at the specified concentration in a 5 mL tube. This mixture was tumbled overnight at 4 °C to facilitate adsorption one day prior to immunization. Rabbit immunization experiments were carried out at Cocalico Biologicals Inc. (Stevens, Hoboken, NJ, USA). All animal experiments were approved by the local Institutional Animal Care and Use Committee.

Enzyme-linked immunosorbent assay (ELISA). IgG antibody titers against *S.* Typhimurium OSP, *S.* Enteritidis OSP, *S.* Typhi Vi, *S.* Paratyphi A OSP, and the fusion proteins IpaB, SseB, and CP1 were measured using the methods described previously [23,24]. Briefly, NUNC Maxisorp plates were coated with 100 µL of 1 µg/mL (protein) or 10 µg/mL (polysaccharide) in PBS overnight at room temperature. Plates were washed with PBS Tween (PBST) and then blocked with 200 µL of PBS 1% BSA for 1 h. Serum was serially (1:3, starting from 1:50) diluted in 100 µL of PBST and incubated for 2 h at room temperature. After washing, secondary antibody (anti-rabbit IgG Fc HRP conjugate (Jackson ImmunoResearch Catalog #111-035-046)) was diluted 1:10,000 in PBST and incubated for 1 h. Plates were washed and developed with Sureblue. The reaction was stopped with 1 M HCL and read at 450 nM.

Opsonophagocytic assays (OPAs). *S.* Typhi OPAs was performed as has been described previously [20,24,25], with *S*. Typhimurium strains carrying an empty vector (Strain C5) or expressing Vi polysaccharide on the surface (Strain C5.507) [23]. The quadrivalent sera were absorbed with 25 µg/mL of *S.* Typhimurium OSP to eliminate the background killing of the C5.507 strain by anti-Typhimurium antibody before the assay. Activity of antisera against *S.* Typhimurium and *S.* Enteritidis was also analyzed via OPAs. Briefly, these assays were performed by first incubating target bacteria with the dilutions of heat-inactivated rabbit serum to allow the antibody to bind, after which differentiated HL60 cells and complement were incubated with the bacteria and antibodies. The reaction was terminated, and samples were plated on blood agar plates. Colonies were counted to represent the bacteria that had survived the killing. The inverse of the lowest concentration of serum in which 50% of killing occurred was defined as the killing titer.

Bactericidal assays. SBAs for *S*. Paratyphi were carried out as has been described previously, using the ATCC 9150 strain [26]. Bactericidal assays were set up in the same manner as the opsonophagocytic assays described above, except that differentiated HL60s were not used. The killing titer analysis is the same as for OPAs.

## 3. Results 

### 3.1. Comparison of Different Fusion Proteins in *S.* Enteritidis MAPS Complex

**Figure 1:**
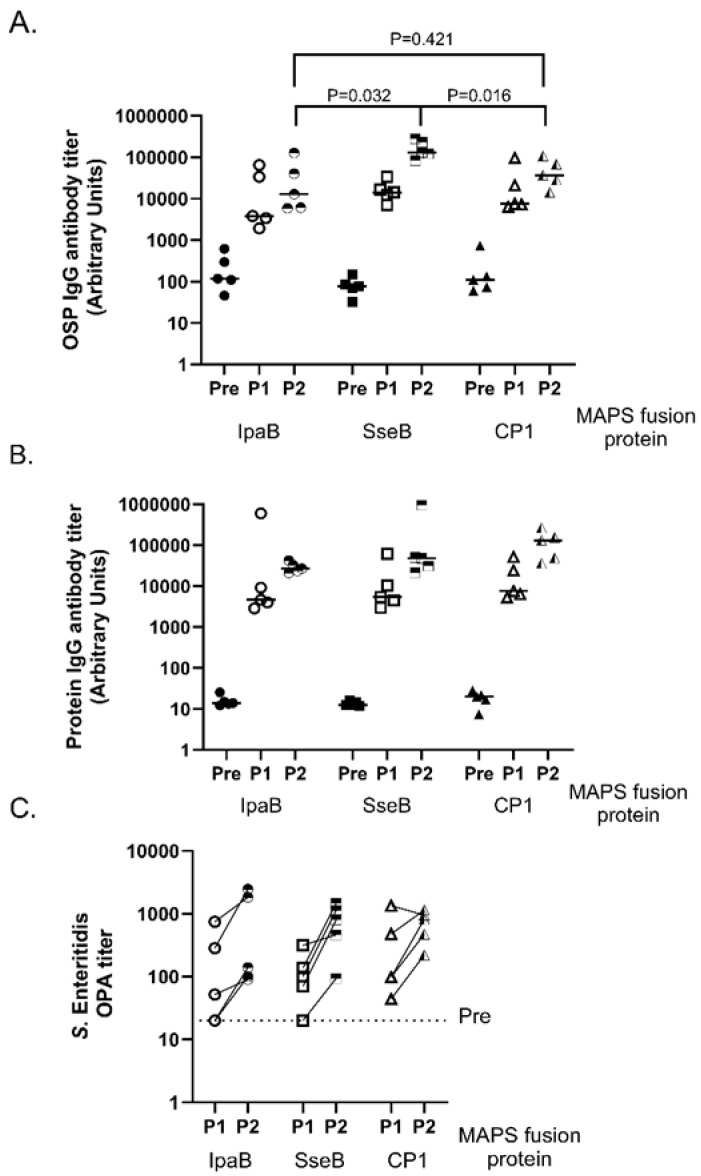
Comparison of three fusion proteins in *Salmonella* Enteritidis MAPS. Pre: rabbit sera before MAPS immunization (closed symbols). P1: rabbit sera after one immunization (open symbols). P2: rabbit sera after two immunizations (half-filled symbols). (**A**) *S.* Enteritidis OSP IgG antibody titer from rabbits immunized with MAPS constructs to compare carrier function of each fusion protein. (**B**) Analysis of IgG antibody against IpaB, SseB, and CP1 from rabbits immunized with MAPS constructs. (**C**). Analysis of IgG antibody function generated by MAPS immunization. Opsonophagocytic assays using P1 and P2 sera were used to determine the killing titers for each rabbit. Data were collected from two or more experiments; a representation is shown here.

Three fusion proteins were tested to assess which was most immunogenic with respect to the *Salmonella* OSP from *S.* Enteritidis. In addition to Rhavi-SseB, we also included Rhavi-IpaB (an analog of SseB in all *Shigella* species), as well as CP1 (Rhavi-SP1500-SP0785), which was used previously for the bivalent Typhi and Paratyphi A vaccine [1]. MAPS constructs were formulated at a 4:1 protein-to-polysaccharide ratio. Each immunization contained 5 µg polysaccharide/dose. In Figure 1A, the three MAPS vaccines were administered to rabbits in two doses spaced three weeks apart. Blood sera were collected at three different time points: before the initial injection (pre, solid symbols), three weeks after the first injection (P1, open symbols), and three weeks after the second injection (P2, half-open symbols). ELISAs against the two antigens of the vaccine (OSP and relevant protein) were performed to analyze serum IgG antibody titers.

Rabbits that received any of the three MAPS constructs had approximately a 1000-fold increase in antibody titer to the OSP compared to baseline after two doses (Figure 1A). Rabbits that received SseB-*S.* Enteritidis MAPS vaccines had the highest average antibody titers, statistically significantly higher than titers from those immunized with MAPS vaccines, including Rhavi-IpaB (*p* = 0.032) or CP1 (*p* = 0.016). There was no statistically significant difference in the response to vaccines containing Rhavi-IpaB and CP1. All three fusion proteins were immunogenic, as expected (Figure 1B).

Next, we evaluated functional antibody responses. Opsonophagocytic assays (OPAs), instead of bactericidal assays, were used to assess the functionality of MAPS-induced antibodies with *S.* Enteritidis OSP due to its reproducibility across different isolates (Figure 1C) [27]. Immunized rabbit sera were assessed post one (P1) and post two (P2) immunizations for their ability to opsonize and enable phagocytic killing of *S.* Enteritidis bacterial cells (Figure 1C). Rhavi-SseB-*S.* Enteritidis MAPS generated a similar killing titer after two immunizations (Figure 1C, squares) compared to *S.* Enteritidis MAPS associated with Rhavi-IpaB or CP1. Rhavi-SseB-*S.* Enteritidis MAPS-immunized sera demonstrated a similar increase in killing activity between the first and second doses, which suggests that two doses are necessary for this vaccine to be most effective. Of note, we observed positive correlations between the ELISA titer and killing titers of the antisera for *S*. Typhimurium (Spearman test r = 0.9524, *p* = 0.0011) and *S*. Enteritidis (Spearman test r = 0.8333, *p* = 0.015) MAPS after two immunizations (Figure S1).

We conclude that, of the three fusion proteins evaluated, Rhavi-SseB resulted in the highest immune response induced by *Salmonella* MAPS vaccines. Based on this, we conducted all subsequent studies using Rhavi-SseB as the fusion protein. 

### 3.2. Role of Fusion Protein Rhavi-SseB

**Figure 2:**
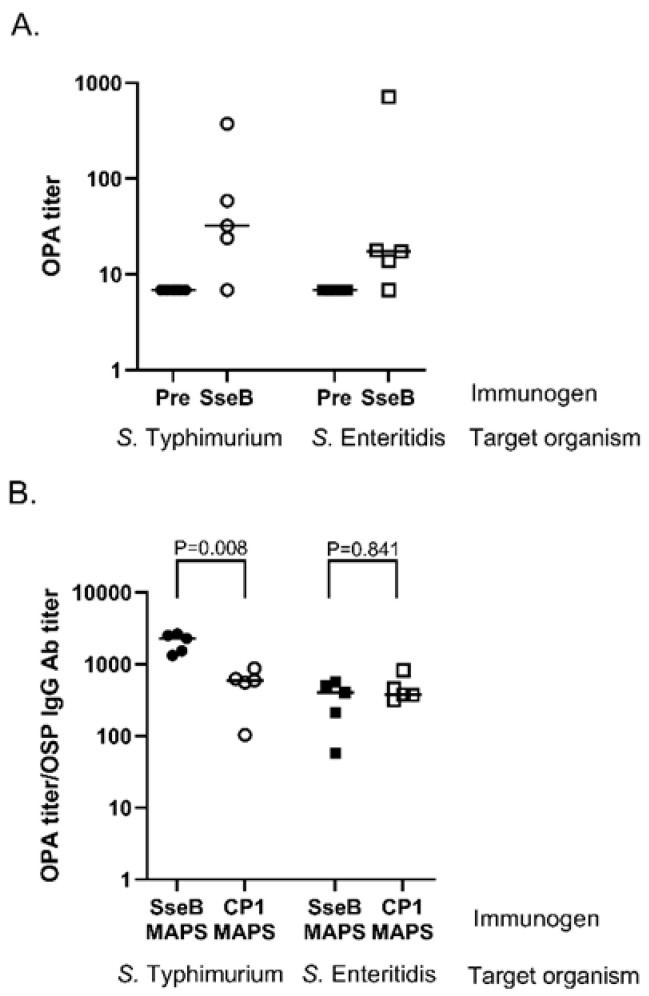
Role of SseB in bacterial killing during opsonophagocytic assays. (**A**) Analysis of the killing capability of SseB-immunized rabbit sera against *S.* Typhimurium (circles) and *S.* Enteritidis (squares). Pre: rabbit sera before MAPS immunization (closed symbols). SseB: sera from rabbits immunized with SseB protein alone (open symbols). (**B**) Protection comparison of SseB MAPS compared with CP1 MAPS against *S.* Typhimurium (circles) and *S.* Enteritidis (squares) in opsonophagocytic assays. Killing titers were divided by OSP antibody titers to normalize the killing activity per antibody. Data were collected from two or more experiments; a representation is shown here.

A potential benefit of using a *Salmonella*-specific protein as the fusion protein is the ability to provide additional (polysaccharide-independent) protection against infection. To evaluate this possibility, we studied the ability of protein-directed antibodies to kill the organism. Rabbits were immunized twice with 100 µg protein of Rhavi-SseB on a two-week schedule. OPAs were performed with the post two immunizations sera and *S.* Typhimurium and *S.* Enteritidis as the target bacteria (Figure 2A, open symbols). We observed killing activity by Rhavi-SseB sera compared to pre-immunization sera (Figure 2A, closed symbols) for both bacteria, demonstrating that antibodies to Rhavi-SseB could provide some additional protective activity against the two *Salmonella* serovars. 

We then tested the function of protein-directed antibodies generated following immunization with MAPS vaccines, using sera from animals immunized with MAPS vaccines made with fusion protein CP1 for comparison. As shown previously, rabbits immunized with SseB-containing MAPS vaccines generated higher antibody concentrations to the polysaccharides than did those with CP1 as fusion protein. To adjust for this, we compared the killing activity in OPA per arbitrary unit of OSP antibody (Figure 2B). For *S.* Typhimurium, the results demonstrated that the killing titer per unit of OSP antibody was significantly higher in sera from Rhavi-SseB MAPS-immunized rabbits compared to those immunized with CP1 MAPS (*p* = 0.008). However, such a difference was not noted when killing activity against *S.* Enteritidis was analyzed in the same fashion. These data suggest that a pathogen-specific fusion protein may enhance killing activity of sera from immunized animals for certain organisms.

### 3.3. Analysis of S. Typhimurium MAPS

**Figure 3:**
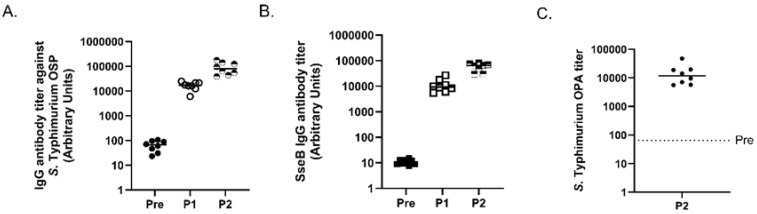
Analysis of *S.* Typhimurium MAPS immunogenicity. (**A**) Analysis of antibody production against *S.* Typhimurium OSP after two MAPS immunizations. Pre: rabbit sera before MAPS immunization (closed symbols). P1: rabbit sera after one immunization (open symbols). P2: rabbit sera after two immunizations (half-filled symbols). (**B**) Analysis of SseB antibody production after immunization with *S.* Typhimurium MAPS vaccines. Pre: rabbit sera before MAPS immunization (closed symbols). P1: rabbit sera after one immunization (open symbols). P2: rabbit sera after two immunizations (half-filled symbols). (**C**) Analysis of killing activity of immunized rabbit sera after two doses of *S.* Typhimurium MAPS vaccines. Depicted are the inverses of the sera dilutions in which a minimum of 50% of the bacteria were killed. The dotted line indicates the killing activity of rabbit sera prior to immunization. Data were collected from two or more experiments; a representation is shown here.

Monovalent MAPS constructs were generated using an OSP from *S.* Typhimurium and Rhavi-SseB as the fusion protein. First, we assessed the production of antibodies against the OSP in rabbits immunized with Rhavi-SseB-*S.* Typhimurium MAPS construct after the first and second immunizations. After two immunizations, the rabbits generated a robust antibody response to *S.* Typhimurium OSP (Figure 3A). The antibody response to the fusion protein Rhavi-SseB was also measured after two immunizations, and similar antibody titers were individually generated by the Rhavi-SseB-*S.* Typhimurium MAPS construct compared to the Rhavi-SseB-*S.* Enteritidis MAPS construct in Figure 1 (Figure 3B). The functionality of the OSP antibodies present after two doses of MAPS vaccines was then tested with opsonophagocytic assays. *S.* Typhimurium MAPS vaccines generated antibodies with robust killing activity (Figure 3C). Together, the data from Figure 1 and Figure 3 show how both MAPS constructions produced potent antibodies against the vaccine’s antigens and showed the ability to eradicate bacteria carrying the same OSP.

### 3.4. Multivalent MAPS against Two and Four Salmonella Species

To target several clinically relevant *Salmonella* species in a single vaccine, multivalent MAPS vaccines were constructed by incorporating monovalent MAPS. We tested a bivalent MAPS construct (containing *S.* Typhimurium and *S.* Enteritidis OSP) and a quadrivalent MAPS (containing *S.* Typhimurium, *S.* Enteritidis, *S.* Paratyphi A OSP, and *S.* Typhi Vi) both using Rhavi-SseB as the fusion protein. 

**Figure 4:**
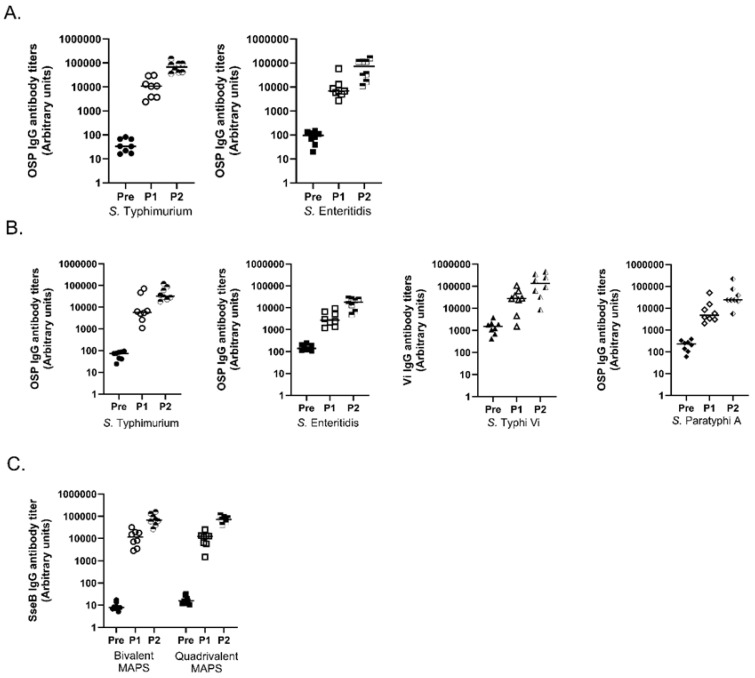
IgG antibody production by multivalent MAPS. (**A**) Analysis of OSP IgG antibody of Bivalent SseB *S.* Typhimurium and *S.* Enteritidis MAPS constructs. (**B**) Analysis of quadrivalent SseB MAPS antibody production against polysaccharide from four *Salmonella* serovars. (**C**) Analysis of SseB antibody production for bivalent and quadrivalent MAPS immunizations. Data were collected from two or more experiments; a representation is shown here. Pre: rabbit sera before MAPS immunization (closed symbols). P1: rabbit sera after one immunization (open symbols). P2: rabbit sera after two immunizations (half-filled symbols).

We tested the antibody production in rabbits immunized with the bivalent MAPS vaccine. Robust antibody responses were generated against both OSPs, at similar levels to those achieved by each monovalent MAPS vaccine (Figure 4A compared to Figure 1A and Figure 3A). These results suggest little to no interference in antibody production with the addition of another MAPS construct in the vaccine. We then assessed OSP antibody production by the quadrivalent MAPS vaccine formulation. After two doses of the quadrivalent MAPS vaccine, robust antibody titers were detected for all four polysaccharides (Figure 4B). Both multivalent MAPS vaccines showed high levels of antibody production which were similar to those for Rhavi-SseB after two doses (Figure 4C).

**Figure 5:**
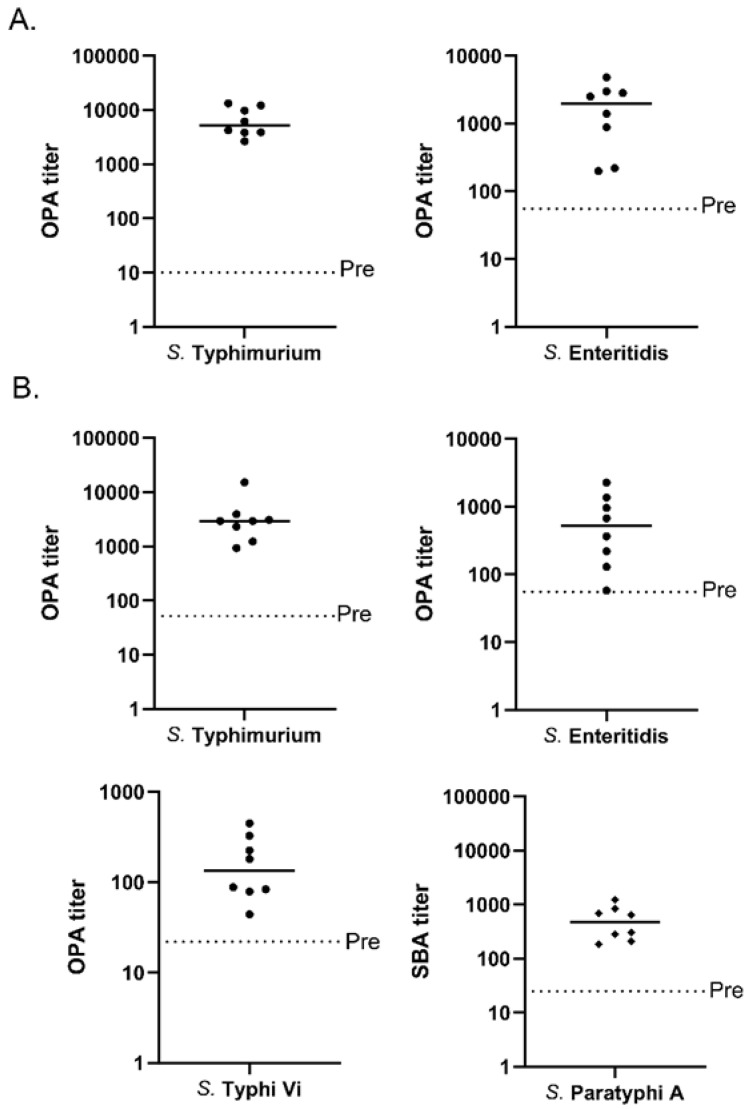
Antibody function analysis of multivalent MAPS constructs after two rabbit immunizations. The inverse of the sera dilution in which 50% of the bacteria were killed is shown. (**A**) Antibody functionality analysis of bivalent MAPS immunizations against *S.* Typhimurium and *S.* Enteritidis in opsonophagocytic assays. (**B**) Analysis of antibody killing activity against the four *Salmonella* serovars. Opsonophagocytic assays were used to analyze *S.* Typhimurium, *S.* Enteritidis, and *S.* Typhi. Bactericidal assays were used to analyze *S.* Paratyphi. Data were collected from two or more experiments; a representation is shown here.

The ability of vaccine-generated antibodies to target and kill the four *Salmonella* species was tested with opsonophagocytic assays for all organisms except *S.* Paratyphi A, for which we used a bactericidal assay. For the bivalent MAPS vaccine, killing activity against *S.* Typhimurium and *S.* Enteritidis was very similar to the killing titers generated with each of the monovalent MAPS vaccines (Figure 5A compared to Figure 1C and Figure 3C). The quadrivalent MAPS vaccine containing all four polysaccharides was tested for the functionality of all the antibodies generated (Figure 5B). The killing titers that were generated against all four *Salmonella* species were similar to the monovalent and bivalent MAPS vaccines’ counterparts (Figure 5B compared to Figure 1C and Figure 3C) [1]. These data suggest that a quadrivalent MAPS vaccine can generate robust and functional antibodies against all four *Salmonella* strains.

## 4. Discussion

Salmonella is a type of facultative intracellular pathogen demonstrating the capability to survive both extracellularly and within monocytes and macrophages [28]. These attributes present a challenge in preventing Salmonella infections, as the bacteria exhibit a remarkable ability to evade the immune system.

Several vaccine techniques to target iNTS have been tested in preclinical trials (reviewed in [6]). A live-attenuated *S.* Typhimurium strain, WT05, was tested in a Phase 1 clinical trial, in which it elicited an immune response and was well-tolerated in healthy volunteers [29,30]. A potential concern, however, was that the bacteria were shed in the stool for up to 23 days after immunization, which poses a risk of infection in vulnerable people and an increased potential for transfer of genetic material [29,30]. *S*. Typhimurium have also been genetically modified to generate anti-LPS antibodies in mice, with limited fecal shedding [30,31,32,33]. 

Another approach is the use of outer membrane vesicles (OMVs), as, for example, the Generalized Modules for Membrane Antigens (GMMA) platform being used by the GSK Vaccines Institute for Global Health (GVGH) [9,34]. GMMA employs immunogenic, scalable OMV-based components that have been tested in Phase I and Phase II clinical trials for *Shigella sonnei* (NCT02676895 and NCT03527173) [30,35,36,37,38] and are now being evaluated for iNTS in Phase I and II studies (NCT05480800).

Several studies have demonstrated that antibodies against *S.* Typhimurium and *S.* Enteritidis OSP are protective in mice [7,9,10,11,30,38,39,40,41,42,43]. Studies of sera collected from healthy individuals identified antibodies to the OSP of *S*. Typhimurium that had bactericidal activity [44,45,46]. Taken together, these data suggest that eliciting B-cell responses to these antigens may represent a viable vaccine strategy. Experimental O-antigen-based conjugate vaccines of *S.* Typhimurium and *S.* Enteritidis protected mice from lethal *Salmonella* infections [9,41,43,47,48,49]. The Center for Vaccine Development and Global Health has developed a conjugate vaccine for *S.* Typhimurium and *S.* Enteritidis. A vaccine based on core and O-specific polysaccharides (OSPs) chemically linked to flagellin protein FliC was immunogenic and protective in mice and has completed a Phase 1 clinical trial (NCT03981952) [8,30,48]. As a parallel approach, surface-exposed, genetically conserved *Salmonella* proteins have been studied for their potential to generate protection from infection. Members of the outer membrane protein family (OmpCDF), siderophores, and proteins from the type III secretion system are immunogenic in mice [5,50,51,52].

In this work, we designed two MAPS constructs to target *S.* Typhimurium and *S.* Enteritidis using O-specific polysaccharides from both organisms. We determined that the *Salmonella*-specific protein SseB was an excellent fusion protein in these constructs (Figure 1), resulting in high antibody titers to OSP and excellent killing activity. We focused on SseB due to its conserved nature, its location on the cell surface, and its role in infection [5,14,53,54] (Figure 3, Figure 4 and Figure 5). We also observed that SseB can potentially provide additional protection for a strain of *S.* Typhimurium (Figure 2). Our approach has unique features compared to others. In addition to the robust antibody responses to OSP, MAPS also generated functional antibodies against the important Salmonella antigen SseB. However, the role of anti-SseB antibodies in the protection against Salmonella serovar still needs to be confirmed in future clinical trials.

We designed bivalent and quadrivalent *Salmonella* MAPS vaccines and demonstrated that combining MAPS into one immunization can potentially provide protection against multiple organisms. The bivalent MAPS vaccine containing OSPs for *S.* Typhimurium and *S.* Enteritidis demonstrated significant antibody production and killing activity, comparable to their monovalent counterparts (Figure 4 and Figure 5). The quadrivalent MAPS vaccine against *S.* Typhimurium*, S.* Enteritidis*, S.* Typhi Vi*,* and *S.* Paratyphi A induced production of significant functional antibodies (Figure 4 and Figure 5). The data demonstrate that protection could be potentially provided for all four serovars of *Salmonella enterica* with two doses of this vaccine. This would eliminate the need for multiple differing immunizations in endemic areas where vaccine compliance may be difficult [6]. Overall, our data indicate that applying MAPS technology to vaccine development against invasive forms of *Salmonella* is feasible and warrants further consideration and confirmation of protection by challenge studies.

## 5. Conclusions

Our study showed that a combinational MAPS vaccine containing Vi, OSPs from *S.* Typhimurium, *S.* Enteritidis, and *S.* Paratyphi A, and conserved Salmonella protein SseB generated functional antibodies against all the components included in the vaccine. The results support further development of the vaccine.

## Data Availability

Data are available upon request.

## Supplementary Materials

The following supporting information can be downloaded at: https://www.mdpi.com/article/10.3390/vaccines11111671/s1, Figure S1: Correlation between ELISA titer and OPA titer for *S.* Typhimurium (A) and *S.* Enteritidis (B).

## References

[1] Zhang F., Boerth E.M., Gong J., Ma N., Lucas K., Ledue O., Malley R., Lu Y.-J. (2022). A Bivalent MAPS Vaccine Induces Protective Antibody Responses against Salmonella Typhi and Paratyphi A. Vaccines.

[2] Haselbeck A.H., Panzner U., Im J., Baker S., Meyer C.G., Marks F. (2017). Current perspectives on invasive nontyphoidal Salmonella disease. Curr. Opin. Infect. Dis..

[3] Tennant S.M., MacLennan C.A., Simon R., Martin L.B., Khan M.I. (2016). Nontyphoidal salmonella disease: Current status of vaccine research and development. Vaccine.

[4] Hagedoorn N.N., Murthy S., Birkhold M., Marchello C.S., Crump J.A. (2023). Prevalence and distribution of non-typhoidal *Salmonella enterica* serogroups and serovars isolated from normally sterile sites: A global systematic review. medRxiv.

[5] Baliban S.M., Lu Y.J., Malley R. (2020). Overview of the Nontyphoidal and Paratyphoidal Salmonella Vaccine Pipeline: Current Status and Future Prospects. Clin. Infect. Dis..

[6] MacLennan C.A., Stanaway J., Grow S., Vannice K., Steele A.D. (2023). *Salmonella* Combination Vaccines: Moving Beyond Typhoid. Open Forum. Infect. Dis..

[7] Micoli F., Ravenscroft N., Cescutti P., Stefanetti G., Londero S., Rondini S., MacLennan C.A. (2014). Structural analysis of O-polysaccharide chains extracted from different *Salmonella* Typhimurium strains. Carbohydr. Res..

[8] Baliban S.M., Curtis B., Toema D., Tennant S.M., Levine M.M., Pasetti M.F., Simon R. (2018). Immunogenicity and efficacy following sequential parenterally-administered doses of Salmonella Enteritidis COPS:FliC glycoconjugates in infant and adult mice. PLoS Negl. Trop. Dis..

[9] Micoli F., Rondini S., Alfini R., Lanzilao L., Necchi F., Negrea A., Rossi O., Brandt C., Clare S., Mastroeni P. (2018). Comparative immunogenicity and efficacy of equivalent outer membrane vesicle and glycoconjugate vaccines against nontyphoidal Salmonella. Proc. Natl. Acad. Sci. USA.

[10] Schuster O., Sears K.T., Ramachandran G., Fuche F.J., Curtis B., Tennant S.M., Simon R. (2019). Immunogenicity and protective efficacy against Salmonella C(2)-C(3) infection in mice immunized with a glycoconjugate of S. Newport Core-O polysaccharide linked to the homologous serovar FliC protein. Hum. Vaccin. Immunother..

[11] Goh Y.S., Clare S., Micoli F., Saul A., Mastroeni P., MacLennan C.A. (2015). Monoclonal Antibodies of a Diverse Isotype Induced by an O-Antigen Glycoconjugate Vaccine Mediate In Vitro and In Vivo Killing of African Invasive Nontyphoidal Salmonella. Infect. Immun..

[12] Ruiz-Albert J., Mundy R., Yu X.J., Beuzón C.R., Holden D.W. (2003). SseA is a chaperone for the SseB and SseD translocon components of the Salmonella pathogenicity-island-2-encoded type III secretion system. Microbiology.

[13] Reynolds C.J., Jones C., Blohmke C.J., Darton T.C., Goudet A., Sergeant R., Maillere B., Pollard A.J., Altmann D.M., Boyton R.J. (2014). The serodominant secreted effector protein of Salmonella, SseB, is a strong CD4 antigen containing an immunodominant epitope presented by diverse HLA class II alleles. Immunology.

[14] Lee S.J., Liang L., Juarez S., Nanton M.R., Gondwe E.N., Msefula C.L., Kayala M.A., Necchi F., Heath J.N., Hart P. (2012). Identification of a common immune signature in murine and human systemic Salmonellosis. Proc. Natl. Acad. Sci. USA.

[15] Barat S., Willer Y., Rizos K., Claudi B., Mazé A., Schemmer A.K., Kirchhoff D., Schmidt A., Burton N., Bumann D. (2012). Immunity to intracellular Salmonella depends on surface-associated antigens. PLoS Pathog..

[16] Kang X., Huang T., Shen H., Meng C., Jiao X., Pan Z. (2022). Salmonella Enteritidis Subunit Vaccine Candidate Based on SseB Protein Co-Delivered with Simvastatin as Adjuvant. Pathogens.

[17] Desin T.S., Köster W., Potter A.A. (2013). *Salmonella vaccines* in poultry: Past, present and future. Expert. Rev. Vaccines.

[18] Zhang F., Lu Y.J., Malley R. (2013). Multiple antigen-presenting system (MAPS) to induce comprehensive B- and T-cell immunity. Proc. Natl. Acad. Sci. USA.

[19] Chichili G.R., Smulders R., Santos V., Cywin B., Kovanda L., Van Sant C., Malinoski F., Sebastian S., Siber G., Malley R. (2022). Phase 1/2 study of a novel 24-valent pneumococcal vaccine in healthy adults aged 18 to 64 years and in older adults aged 65 to 85 years. Vaccine.

[20] Szu S.C., Li X.R., Schneerson R., Vickers J.H., Bryla D., Robbins J.B. (1989). Comparative immunogenicities of Vi polysaccharide-protein conjugates composed of cholera toxin or its B subunit as a carrier bound to high- or lower-molecular-weight Vi. Infect. Immun..

[21] Micoli F., Rondini S., Gavini M., Pisoni I., Lanzilao L., Colucci A., Giannelli C., Pippi F., Sollai L., Pinto V. (2013). A scalable method for O-antigen purification applied to various Salmonella serovars. Anal. Biochem..

[22] Roe J.H. (1955). The determination of sugar in blood and spinal fluid with anthrone reagent. J. Biol. Chem..

[23] Konadu E., Shiloach J., Bryla D.A., Robbins J.B., Szu S.C. (1996). Synthesis, characterization, and immunological properties in mice of conjugates composed of detoxified lipopolysaccharide of Salmonella paratyphi A bound to tetanus toxoid with emphasis on the role of O acetyls. Infect. Immun..

[24] Lu Y.-J., Zhang F., Sayeed S., Thompson C.M., Szu S., Anderson P.W., Malley R. (2012). A bivalent vaccine to protect against Streptococcus pneumoniae and Salmonella typhi. Vaccine.

[25] Hale C., Bowe F., Pickard D., Clare S., Haeuw J.-F., Powers U., Menager N., Mastroeni P., Dougan G. (2006). Evaluation of a novel Vi conjugate vaccine in a murine model of salmonellosis. Vaccine.

[26] Boyd M.A., Tennant S.M., Saague V.A., Simon R., Muhsen K., Ramachandran G., Cross A.S., Galen J.E., Pasetti M.F., Levine M.M. (2014). Serum bactericidal assays to evaluate typhoidal and nontyphoidal Salmonella vaccines. Clin. Vaccine Immunol..

[27] Gondwe E.N., Molyneux M.E., Goodall M., Graham S.M., Mastroeni P., Drayson M.T., MacLennan C.A. (2010). Importance of antibody and complement for oxidative burst and killing of invasive nontyphoidal Salmonella by blood cells in Africans. Proc. Natl. Acad. Sci. USA.

[28] Gilchrist J.J., MacLennan C.A. (2019). Invasive Nontyphoidal Salmonella Disease in Africa. EcoSal Plus.

[29] Hindle Z., Chatfield S.N., Phillimore J., Bentley M., Johnson J., Cosgrove C.A., Ghaem-Maghami M., Sexton A., Khan M., Brennan F.R. (2002). Characterization of Salmonella enterica derivatives harboring defined aroC and Salmonella pathogenicity island 2 type III secretion system (ssaV) mutations by immunization of healthy volunteers. Infect. Immun..

[30] Sears K.T., Galen J.E., Tennant S.M. (2021). Advances in the development of Salmonella-based vaccine strategies for protection against Salmonellosis in humans. J. Appl. Microbiol..

[31] Allam U.S., Krishna M.G., Lahiri A., Joy O., Chakravortty D. (2011). Salmonella enterica serovar Typhimurium lacking hfq gene confers protective immunity against murine typhoid. PLoS ONE.

[32] Wang Y., Li J., Xiong K., Chen Z., Zheng C., Tan Y., Cong Y. (2017). Elimination of persistent vaccine bacteria of Salmonella enterica serovar Typhimurium in the guts of immunized mice by inducible expression of truncated YncE. PLoS ONE.

[33] El Ghany M.A., Jansen A., Clare S., Hall L., Pickard D., Kingsley R.A., Dougan G. (2007). Candidate live, attenuated Salmonella enterica serotype Typhimurium vaccines with reduced fecal shedding are immunogenic and effective oral vaccines. Infect. Immun..

[34] De Benedetto G., Alfini R., Cescutti P., Caboni M., Lanzilao L., Necchi F., Saul A., MacLennan C., Rondini S., Micoli F. (2017). Characterization of O-antigen delivered by Generalized Modules for Membrane Antigens (GMMA) vaccine candidates against nontyphoidal Salmonella. Vaccine.

[35] Launay O., Lewis D.J., Anemona A., Loulergue P., Leahy J., Sciré A.S., Maugard A., Marchetti E., Zancan S., Huo Z. (2017). Safety Profile and Immunologic Responses of a Novel Vaccine Against Shigella sonnei Administered Intramuscularly, Intradermally and Intranasally: Results from Two Parallel Randomized Phase 1 Clinical Studies in Healthy Adult Volunteers in Europe. EBioMedicine.

[36] Obiero C.W., Ndiaye A.G.W., Sciré A.S., Kaunyangi B.M., Marchetti E., Gone A.M., Schütte L.D., Riccucci D., Auerbach J., Saul A. (2017). A Phase 2a Randomized Study to Evaluate the Safety and Immunogenicity of the 1790GAHB Generalized Modules for Membrane Antigen Vaccine against Shigella sonnei Administered Intramuscularly to Adults from a Shigellosis-Endemic Country. Front. Immunol..

[37] Gerke C., Colucci A.M., Giannelli C., Sanzone S., Vitali C.G., Sollai L., Rossi O., Martin L.B., Auerbach J., Di Cioccio V. (2015). Production of a Shigella sonnei Vaccine Based on Generalized Modules for Membrane Antigens (GMMA), 1790GAHB. PLoS ONE.

[38] Baliban S.M., Allen J.C., Curtis B., Amin M.N., Lees A., Rao R.N., Naidu G., Venkatesan R., Rao D.Y., Mohan V.K. (2018). Immunogenicity and Induction of Functional Antibodies in Rabbits Immunized with a Trivalent Typhoid-Invasive Nontyphoidal Salmonella Glycoconjugate Formulation. Molecules.

[39] Li P., Liu Q., Luo H., Liang K., Yi J., Luo Y., Hu Y., Han Y., Kong Q. (2017). O-Serotype Conversion in Salmonella Typhimurium Induces Protective Immune Responses against Invasive Non-Typhoidal Salmonella Infections. Front. Immunol..

[40] Liu Q., Li P., Luo H., Curtiss R., Kong Q. (2019). Attenuated Salmonella Typhimurium expressing Salmonella Paratyphoid A O-antigen induces protective immune responses against two Salmonella strains. Virulence.

[41] Watson D.C., Robbins J.B., Szu S.C. (1992). Protection of mice against Salmonella typhimurium with an O-specific polysaccharide-protein conjugate vaccine. Infect. Immun..

[42] Jörbeck H.J., Svenson S.B., Lindberg A.A. (1981). Artificial Salmonella vaccines: Salmonella typhimurium O-antigen-specific oligosaccharide-protein conjugates elicit opsonizing antibodies that enhance phagocytosis. Infect. Immun..

[43] Rondini S., Micoli F., Lanzilao L., Gavini M., Alfini R., Brandt C., Clare S., Mastroeni P., Saul A., MacLennan C.A. (2015). Design of glycoconjugate vaccines against invasive African Salmonella enterica serovar Typhimurium. Infect. Immun..

[44] MacLennan C.A., Tennant S.M. (2013). Comparing the roles of antibodies to nontyphoidal Salmonella enterica in high- and low-income countries and implications for vaccine development. Clin. Vaccine Immunol..

[45] Trebicka E., Jacob S., Pirzai W., Hurley B.P., Cherayil B.J. (2013). Role of antilipopolysaccharide antibodies in serum bactericidal activity against Salmonella enterica serovar Typhimurium in healthy adults and children in the United States. Clin. Vaccine Immunol..

[46] Goh Y.S., Necchi F., O’shaughnessy C.M., Micoli F., Gavini M., Young S.P., Msefula C., Gondwe E.N., Mandala W.L., Gordon M. (2016). Bactericidal Immunity to Salmonella in Africans and Mechanisms Causing Its Failure in HIV Infection. PLoS Neglected Trop. Dis..

[47] MacLennan C.A., Martin L.B., Micoli F. (2014). Vaccines against invasive Salmonella disease: Current status and future directions. Hum. Vaccin. Immunother..

[48] Simon R., Tennant S.M., Wang J.Y., Schmidlein P.J., Lees A., Ernst R.K., Pasetti M.F., Galen J.E., Levine M.M. (2011). Salmonella enterica serovar enteritidis core O polysaccharide conjugated to H:g,m flagellin as a candidate vaccine for protection against invasive infection with *S. enteritidis*. Infect. Immun..

[49] Ahmed A., Akhade A.S., Qadri A. (2020). Accessibility of O Antigens Shared between Salmonella Serovars Determines Antibody-Mediated Cross-Protection. J. Immunol..

[50] Lee S.-J., Benoun J., Sheridan B.S., Fogassy Z., Pham O., Pham Q.-M., Puddington L., McSorley S.J. (2017). Dual Immunization with SseB/Flagellin Provides Enhanced Protection against Salmonella Infection Mediated by Circulating Memory Cells. J. Immunol..

[51] Cunningham A.F., Gaspal F., Serre K., Mohr E., Henderson I.R., Scott-Tucker A., Kenny S.M., Khan M., Toellner K.M., Lane P.J. (2007). Salmonella induces a switched antibody response without germinal centers that impedes the extracellular spread of infection. J. Immunol..

[52] Gil-Cruz C., Bobat S., Marshall J.L., Kingsley R.A., Ross E.A., Henderson I.R., Leyton D.L., Coughlan R.E., Khan M., Jensen K.T. (2009). The porin OmpD from nontyphoidal Salmonella is a key target for a protective B1b cell antibody response. Proc. Natl. Acad. Sci. USA.

[53] Martinez-Becerra F.J., Kumar P., Vishwakarma V., Kim J.H., Arizmendi O., Middaugh C.R., Picking W.D., Picking W.L. (2018). Characterization and Protective Efficacy of Type III Secretion Proteins as a Broadly Protective Subunit Vaccine against Salmonella enterica Serotypes. Infect. Immun..

[54] Liu Q., Yi J., Liang K., Liu T., Roland K.L., Jiang Y., Kong Q. (2016). Outer membrane vesicles derived from Salmonella Typhimurium mutants with truncated LPS induce cross-protective immune responses against infection of Salmonella enterica serovars in the mouse model. Int. J. Med. Microbiol..

