# Supplementary figures and images for: Induction of Broad Immunity against Invasive Salmonella Disease by a Quadrivalent Combination Salmonella MAPS Vaccine Targeting Salmonella Enterica Serovars Typhimurium, Enteritidis, Typhi, and Paratyphi A
Source: Vaccines (Basel). 2023 Oct 31;11(11):1671. doi: 10.3390/vaccines11111671 (PMC10675568; doi:10.3390/vaccines11111671)

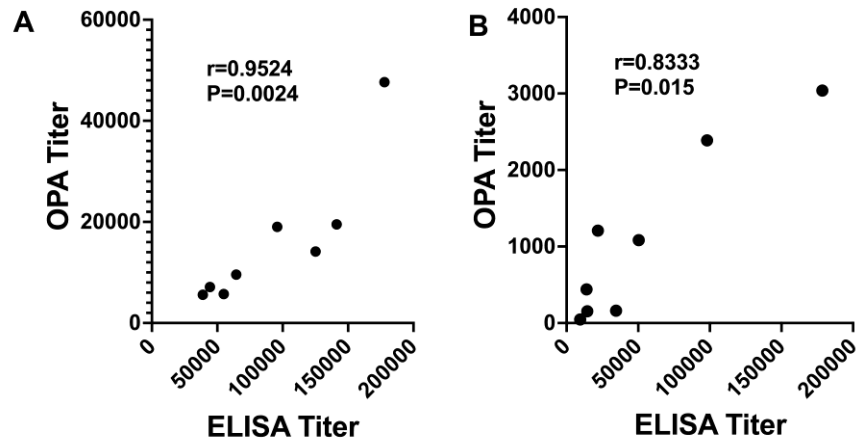

Figure S1. Correlation between ELISA titer and OPA titer for *S. Typhimurium* (A) and *S. Enteritidis* (B).

Supplement: Supplementary file 1 [file vaccines-11-01671-s001.zip › vaccines-2665778-supplementary.pdf]
